# What Ecological Factors Shape Species-Area Curves in Neutral Models?

**DOI:** 10.1371/journal.pone.0038232

**Published:** 2012-06-04

**Authors:** Massimo Cencini, Simone Pigolotti, Miguel A. Muñoz

**Affiliations:** 1 Istituto dei Sistemi Complessi, Consiglio Nazionale delle Ricerche, Rome, Italy; 2 Departament de Fisica i Enginyeria Nuclear, Universitat Politecnica de Catalunya Rambla Sant Nebridi s/n, Terrassa, Barcelona, Spain; 3 Instituto Carlos I de Fsica Teórica y Computacional, Facultad de Ciencias, Universidad de Granada, Granada, Spain; University of Leeds, United Kingdom

## Abstract

Understanding factors that shape biodiversity and species coexistence across scales is of utmost importance in ecology, both theoretically and for conservation policies. Species-area relationships (SARs), measuring how the number of observed species increases upon enlarging the sampled area, constitute a convenient tool for quantifying the spatial structure of biodiversity. While general features of species-area curves are quite universal across ecosystems, some quantitative aspects can change significantly. Several attempts have been made to link these variations to ecological forces. Within the framework of spatially explicit neutral models, here we scrutinize the effect of varying the local population size (i.e. the number of individuals per site) and the level of habitat saturation (allowing for empty sites). We conclude that species-area curves become shallower when the local population size increases, while habitat saturation, unless strongly violated, plays a marginal role. Our findings provide a plausible explanation of why SARs for microorganisms are flatter than those for larger organisms.

## Introduction

Species-area laws quantify the relation between area and the number of species found in that area and represent one of the most robust biodiversity patterns [1]. Clearly, larger areas harbor a greater number of species, but the increase occurs in a remarkably orderly way [2]. Typically, empirical species-area curves display an inverted *S*-shape: at small (local) and very large (continental) areas (*A*) the number of species (*S*) increases in a relatively steep (nearly linear) way with the area, while the increase is shallower at intermediate areas [3]–[5]. Whilst the two extreme regimes are relatively easy to rationalize, the intermediate one remains intriguing and has attracted much attention (see [1] and references therein). Several fitting formulas have been proposed to describe collected data [4], [6], among which, the most widely adopted are the logarithmic law 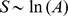 [7] and the power-law relation [8].
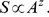
(1)


Data from many field studies tend to slightly favor the power law fit (1) with values of the exponent *z* showing a dependence on environmental variables, e.g., the latitude [1]. Moreover, body-size seems to be an important factor in shaping SARs: with some provisos on possible biases due to undersampling or taxa identification [9], [10], species-area curves for microorganisms are typically shallower than those of larger organisms [11]–[13]. Different hypothesis have been put forward for the reduced spatial diversification of microorganisms (see the review [10] and references therein): enhanced dispersal rates due to large population sizes and short generation times, decreased local diversification due to low extinction rates (owing to large population sizes), and to low speciation rates (because of horizontal gene transfer and imperfect isolation). Despite the role of the local population size in determining the mechanisms above, the effect of its variations has not been tested (to the best of our knowledge) in the context of individual based models.

Along with empirical studies, theoretical efforts have been devoted to identify ecological mechanisms responsible for shaping species-area curves [4]. Examples of these mechanisms include trade-off and interspecific competition [14], [15], or predator-prey dynamics [16] (see [17] for a review). The *Neutral theory* [5] emphasizes the role of *stochastic* mechanisms such as demographic processes, able by themselves to generate nontrivial diversity patterns. In particular, neutral models incorporate processes such as colonization, dispersal, and speciation and assume, in contrast with the *niche* paradigm [18], that all individuals, regardless of the species they belong to, have the same prospects of death, reproduction, etc.

*Spatially implicit* neutral models have been shown to produce species abundance distributions (SADs) in remarkable good agreement with empirical data [5], [19]. This suggests that they capture the essence of general and robust community-level properties or, at least, promotes neutral theories to suitable null-models [20]. Etienne et al. [21] showed that SADs remain unaltered when breaking Hubbell’s [5] “zero-sum assumption”, postulating that the community size is strictly kept constant by resource saturation. Then, the question arises of whether the spatial distribution of species is equally robust upon modifying other “details” of the underlying neutral theory? (see [22]). If not, what are the relevant ecological mechanisms/forces that, implemented in a neutral model, are relevant for shaping the SARs and thus the value of *z* as. For example, what is the relevance of body-size?

*Spatially explicit* neutral models generate species-area curves very similar qualitatively and, to some extent, quantitatively, to empirical ones. They display power-law behaviors with an exponent *z* in a realistic range [23], [24]. Species-area curves in spatially explicit neutral models are mainly shaped by the interplay of dispersal limitation and speciation [23], [25]. In particular, for finite ranged dispersal kernels, regardless their specific form, the actual value of *z* is mainly determined by the speciation rate [26], [27], which is however difficult (or impossible) to estimate. Sensitive variations of the exponent value, at fixed speciation rate, have been observed when the dispersal process couples distant locations in the ecosystem, e.g. by considering fat tailed distributions [28]. The influence of other factors was investigated by Chave et al. [24] who mainly focused on violations of the neutral assumptions, e.g., by introducing trade-offs.

In this paper, we study the effect of varying the number of individuals that can live at a single ecosystem site on the species-area curves generated by neutral spatial models. We consider two kinds of variations: allowing for large *local population sizes*, by letting each site host many individuals, as appropriate for describing communities of microorganisms connected by dispersal, and allowing for empty sites, i.e. changing the level of *habitat saturation*.

To explore these possibilities, we present extensive simulations of the stepping stone model (SSM) [29],[30], which incorporates variable local population size by increasing the number of allowed individuals per site, and the multispecies (or multitype) contact process (MCP) [31], which is suited to study non saturated habitats. These models have been not thoroughly explored before in the context of spatial neutral theory: in particular, the MCP, discussed by Durrett and Levin [23], was not, to the best of our knowledge, previously simulated. The SSM is popular in the context of population genetics but its predictions for species area laws have not been explored before. To complete the picture, we compare the species-area relationships generated by the above models with those for the multispecies voter model (MVM), which is possibly the most studied spatially explicit neutral model [23], [26], [27]. We remark that the term “voter model” is often used to denote the model with nearest-neighbor dispersal among sites. In this paper, we use the same name also when more general dispersal kernels are considered.

Common to all the above models is that individuals of different species are placed at the sites of a two-dimensional lattice and evolve according to basic demographic processes such as birth, death, migration, and speciation. However, important differences also exist. While the MVM and SSM describe saturated habitats with a constant density of individuals, the MCP describes fragmented systems where the density of individuals is irregular both in space and time, with the presence of gaps. The models differ also in the number of allowed individuals per site. In the MVM and MCP each site can hosts one individual at most, as appropriate to describe large organisms, such as trees. In the SSM, each site represents a local community of *M* individuals, making the model more suitable to describe, e.g., patches of microorganisms connected by migration [32]. Indeed, as discussed by Fenchel and Finlay [33], comparing larger and smaller organisms is essentially equivalent to compare organisms with smaller and with larger population sizes, respectively. For instance, it has been estimated that one gram of typical soil can host 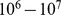 bacteria [34].

We conclude that –together with speciation rate and the dispersion kernel– the size of the local population is an important shaping factor for neutral predictions on species spatial distributions and, hence, on SAR curves. On the other hand, mild violations of habitat saturation – i.e. not as extreme as to break the space into isolated regions – have little effect on the slope of SAR curves on scales larger than the typical size of the gaps.

## Methods

We now present the three aforementioned spatially-explicit neutral models and discuss afterward their main similarities and differences. The section is organized as follows. In the three first subsections we introduce and motivate the models that will be the subject of our study. Then we discuss their similarities, differences and numerical implementation. The last subsection is devoted to a discussion of the effect of the choice of the dispersal kernel.

### Multispecies Voter Model (MVM)

The multispecies voter model is a spatial generalization of the infinite allele Moran model used in population genetics (see, e.g. [35]). Each site of a square lattice is always occupied by a single individual: the habitat is thus saturated. At each time step, a randomly chosen individual on the lattice is killed and immediately replaced: with probability 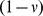, by a randomly chosen copy of one of the nearest neighbors (dispersal event); with probability *v*, by an individual from a new species (speciation event). When 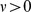, any species will eventually go extinct; speciation events compensate extinctions so that a dynamical equilibrium eventually sets in [23].

### Stepping Stone Model (SSM)

In the previous model, each lattice site hosts a single individual, as appropriate when modeling, e.g. a forest, where each site represents the space occupied by a single tree. In such cases, the limiting resources are indeed strongly related to space, so that it is reasonable to model competition by simply assuming that when an individual dies, a vacant site is left to be occupied by another individual. Conversely, microorganisms, such as small eukaryotes or bacteria, are often present in very large numbers below a scale in which one can assume that all individuals share the same pool of resources. Therefore, it is more appropriate to think of the habitat as subdivided into small patches, connected by migration and each hosting a large population of individuals that directly compete with each other [32], [33]. Such a setting is even more relevant when the habitat is physically divided into patches, so that moving from a patch to another is more difficult than moving within a patch, like in the case of an island chain or of soil fragmented in different soil grains. [36].

In this perspective, the stepping stone model, originally introduced in population genetics [29], straightforwardly generalizes the MVM by allowing each site to host a fixed (but arbitrary) number, *M*, of individuals. At each time step an individual is randomly selected, killed and then replaced: with probability 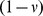 by the offspring of an existing individual or, with probability *v* by an individual of a new species. In the former case, the parent is chosen with probability 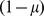 among the remaining 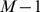 individuals residing at the same site and with probability 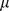 among those at a randomly chosen nearest neighbor site. For 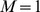, the SSM recovers the MVM with (as detailed in Appendices S1 and S2) *v* substituted by 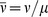, which is the effective speciation-to-diffusion ratio in the SSM. Note that, in general, as dispersal occurs every 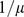 time steps and not at every time step as in the other models, one can show that comparison should be done equating 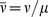 (speciation-to-migration rate) to the value of *v* used in the other models (see, e.g, [30]).

### Multispecies Contact Process (MCP)

In the MVM, gaps left by deaths are immediately filled by newborns leading to habitat saturation. This is tantamount to assuming reproduction rates infinitely larger than death rates [23]. In the contact process [31], this assumption is relaxed and gaps can survive for arbitrarily large times. In particular, each individual dies at rate 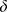 and reproduces at rate 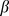 giving rise to a newborn at a randomly chosen neighbor site. As each site cannot host more than one individual, attempted reproduction is successful only if an empty neighbor is chosen. When reproduction is successful, the newborn belongs to the parent species with probability 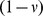 and to a new species with probability *v*. Thus, the relevant parameters are the speciation rate *v* and the birth-to-death ratio 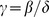, controlling the fraction of occupied sites in steady state conditions. For large 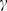, gaps are small and infrequent: in the limit 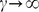 the MCP recovers the MVM [23]. Conversely, lowering 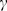 results in an unsaturated habitat with larger and longer-lived gaps. Finally, at 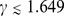, i.e. the CP critical point [37], births become too infrequent, leading to global extinction.

### Similarities and Differences between Models

We now discuss the main similarities and differences among the above models, as summarized in Table 1. A key feature is the maximum number of individuals allowed at each site: 1 for the MVM and the MCP, *M* for the SSM. While the MVM and the SSM describe a saturated habitat, in the MCP, as sites can be empty, the habitat is not saturated. In all models, diversification is implemented as point speciation [5], which of course should not be regarded as a realistic speciation mechanism but rather as an effective one [38]. In this perspective, the speciation rate *v* has to be interpreted as a normalized rate (speciation over death rate). Moreover, as said above, due to the different dispersal rule, in SSM the proper quantity to set up a comparison with the other two models is the speciation to migration ratio 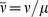.

**Table 1:** Summary of models.

model	local population	saturation
MVM	1	Y
MCP	{0,1}	N
SSM	*M*	Y

Summary of the main features of the considered spatially-explicit neutral models. Y/N stands for Yes/No.

Concerning the simulation scheme, the voter model and the stepping stone model can be reformulated in terms of coalescent random walkers [39], leading to approximate estimates of the exponent *z* (that, for MVM, were put forward in [23], [25]) and also to very efficient numerical implementations [26], [27], [40]. One of the main advantage of this method is that numerical simulations are virtually free from boundary effect problems as if simulating a portion of an infinite landscape [26]. Details on the coalescing random walk analogy, the resulting numerical scheme and analytical estimates are discussed in Appendices S1, S2 and S3. Unfortunately, such reformulation does not easily extend to the multitype contact process, which was simulated by means of a standard algorithm [37] adapted to the multitype case. In this case, periodic boundary conditions have been employed and tests to minimize possible finite size effects performed. Appendix S4 details the numerical scheme.

To close this section, we remind that, while in this paper we restricted our comparison to models in which competitive interactions among individuals are present, recently, O’Dwyer and Green [41] introduced a model in which individuals do not compete (so that species are independent). In this case the number of individuals per site is unrestricted; the advantage of this simplifying assumption is that it allows for a full analytical treatment of the problem.

### Dispersal Kernel and Species-area Relationships

The above models have an additional degree of freedom related to the choice of the dispersal kernel, which is, in general, important to reproduce SAR curves similar to empirical ones. In particular, nearest neighbor (NN) kernels generate biphasic SAR curves rather than triphasic ones [24], because the steep-growth regime at small areas cannot be reproduced. To observe triphasic *S*-shaped SAR, similar to empirical ones, requires more general (finite-range) dispersal kernels, acting on several sites. Moreover, the resulting SAR curves do not depend on the shape of the kernel but only on its range [26]. Fat-tailed dispersal kernels could also be considered to model some dispersal mechanisms found in nature, and have been found to quantitatively influence SARs both in terms of the extension of the intermediate range and in terms of the exponent *z* values [28].

In this paper we mostly explored the behavior of the species-area curves by implementing the above described models with the nearest-neighbor kernel. This choice is mainly dictated by its simplicity and by the costs of simulating the SSM with large lattices (as necessary if more general kernels are used) when the local population size becomes large. Moreover, for the MVM, at small areas SAR curves obtained with NN-kernel approximatively behave as power laws and qualitatively match the behavior of the intermediate (power law) regime of more general kernels [27].

However, to test the robustness of our main findings against the kernel choice we also performed simulations by employing a finite-range square kernel: a killed individual at a given site can be replaced by any of the individuals present in a square centered at that site and having side 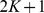. We remind that for the MVM, as soon as 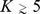, *z* does not depend on *K* and the entire curve can be rescaled [26], [27]. For this reason tests have been performed at 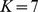.

## Results

The speciation rate *v* determines most features of neutral species-area curves, in particular, the interesting power-law regime Eq. (1) [23], [24], [26], [27], [42]. Therefore, to discriminate the influence of the different ecological mechanisms incorporated in the models, we will compare species-area curves obtained by the above introduced models at equal *v*.

Once *v* (and the dispersal kernel) are fixed, the MVM is fully specified, while the SSM and the MCP need additional parameters to be set. As previously shown, both the SSM and the MCP reduce to the MVM for 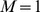 and large birth over death rate ratio (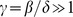), respectively. Hence, to stay away from this limit, we allowed for a large local community size for the SSM by choosing 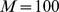 with migration probability 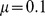 (holding 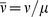 equal to *v* in the other models, as specified above), and considered habitat unsaturated conditions for the MCP by choosing 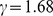, ensuring that only 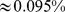 of the available sites are occupied.

**Figure 1:**
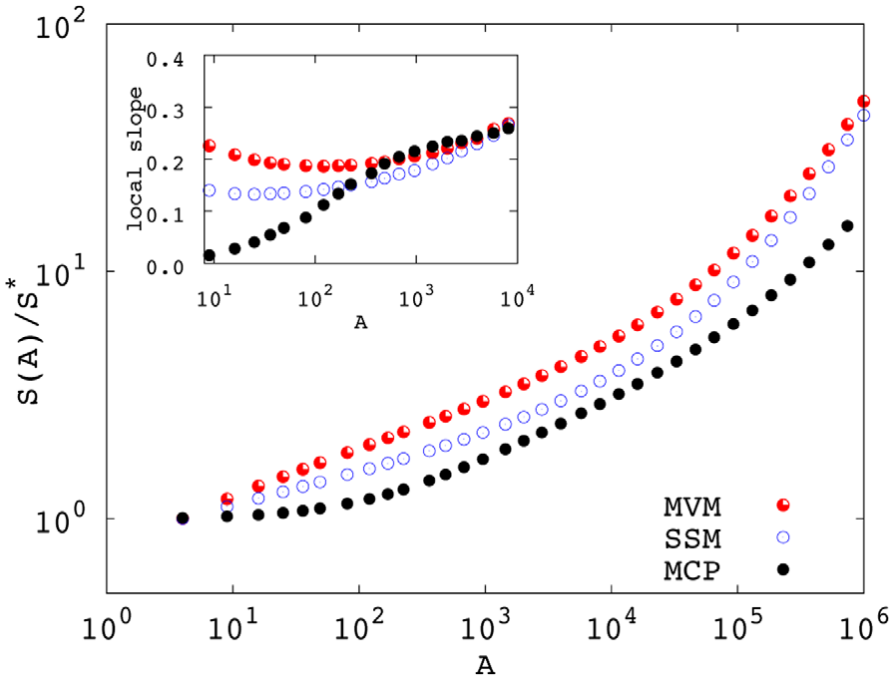
SARs generated by the three models at 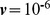 (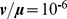 for the SSM) using nearest neighbor dispersal. The MVM and the SSM are simulated on a 1000×1000 square lattice. In the SSM, we chose 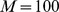 and 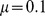. The MCP is simulated on a 2500×2500 lattice with 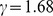 adopting weighted averages (see Fig. 4 and related discussion). In all cases, we averaged over 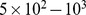 independent realizations and statistical errors are smaller than symbols’ size. To ease the comparison, *S* has been normalized by the average number of species 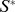 at the smallest sampled area. Inset: local slopes 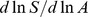 of the four curves for areas smaller than 10^4^.

To compare species-area curves generated by the three models, we performed extensive numerical simulations of the MCP and the SSM (see Appendices S2 and S4 for the numerical implementation). Most of the simulations have been performed by using nearest-neighbor kernels and tests on SSM and MVM have been done using the square kernel discussed in *Methods*. For the MVM we relied on already published numerical results [27]. Figure 1 shows the species-area curves generated by the three models at *v* (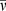 in the SSM) equal to 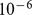. The curves are qualitatively similar to each other. They display a shallower than linear growth for small areas and become steeper, eventually linear, at larger areas. The transition between these two regimes occurs at a similar scale (shown to be 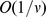 for the MVM [23]) in all models.

The interesting regime can be quantitatively scrutinized by looking at the local slopes, 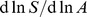, shown in the inset of Fig. 1 for small areas. A power-law range, as in Eq. (1), would correspond to a region in which 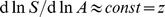. This is a good approximation for the MVM and the SSM whose local slopes are characterized by a shallow parabolic shape. As customary in recent literature [26], [27], [41], in the following we shall determine the exponent *z* as the minimum of this parabola; equivalent (within error bars) results can be obtained fitting a power-law as Eq. (1) on the species-area curve in the scaling range.

Local slopes and thus *z* display some variability among the three models. In particular, the stepping stone model gives rise to shallower curves with respect to the voter model, i.e. 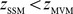. On the other hand, no clear power-law range can be identified for the MCP, as the local slope increases monotonically from zero at increasing the area. We anticipate that this behavior is due to the presence of gaps in the distribution of individuals (see the subsection *Multispecies Contact Process* below).

**Figure 2:**
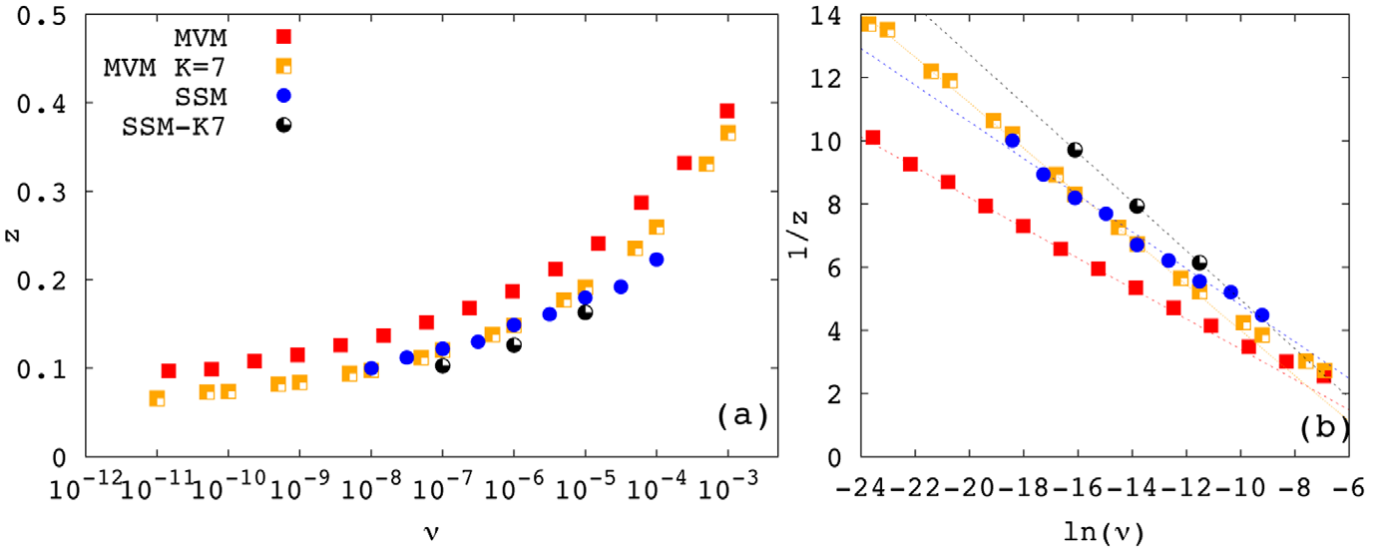
Exponent *z* as a function of *v* for the MVM and SSM. Panel (a) shows *z* vs *v* for MVM and SSM with NN- and square-kernel with *K* = 7. The SSM is simulated for *M* = 100 and 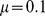. Due to computational limitation, simulations for the SSM at *K* = 7 have been performed at three different values of *v* only. The system size has been chosen in each simulation in order to properly resolve the power-law regime. Panel (b) shows 1/*z* vs 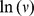 for the same data of (a). Dotted straight lines are best fits obtained using Eq. (2). Fitted values are: for the NN-kernel MVM 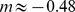 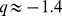 and SSM 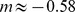 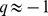; for the square-kernel *K* = 7 MVM 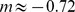 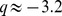 and SSM 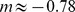 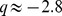.

**Figure 3:**
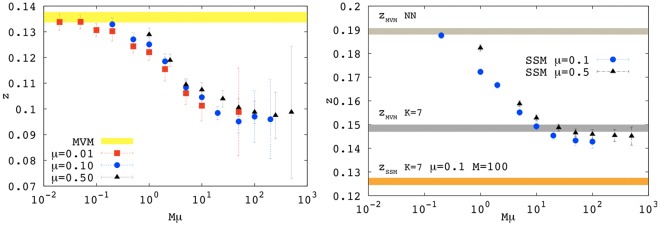
Exponent *z* for the SSM as a function of 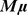 at fixed 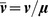. The left panel shows the case 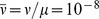 for 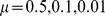 with NN-kernel. The shaded area indicates the value 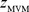 (including the estimated error) of the exponent for the MVM with 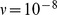. For 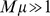 statistical errors increase because a smaller number of realizations was used as simulations become very costly. The right panel shows the case 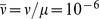 for 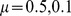 with NN-kernel. The shaded areas display the value of the exponent for the MVM with both the NN- and square-kernel (*K* = 7) and the SSM with square-kernel (*K* = 7) for *M* = 100 and 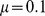 (i.e. 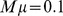).



Figure 2a shows the dependence of the exponent *z* on the speciation rate *v* (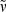 for SSM) for the MVM and SSM; MCP was excluded because as seen in Fig. 1 no reasonable power-law range exists for 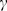 close to 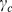. Let us start comparing the two models with NN dispersal. As for the case 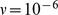 (Fig. 1), the exponents are different and the curves produced by the SSM are consistently shallower than those generated by the voter model in the explored range of *v*-values. In this figure we can see that the exponents for the SSM with NN-dispersal appear to be close to (but not coincident with) those of the MVM with the square-kernel (*K* = 7). However, when comparing the exponents of the SSM and MVM when the square-kernel (*K* = 7) is employed for both, we still observe that the former is shallower (see also Fig 3 and its discussion in the next section). Notice that increasing further *K* in the MVM does not produce further changes in the exponent [26], [27]. Therefore, as the comparison with the same dispersal kernel reveals, the decrease in the exponent *z* due to the increase of the local population size is a genuine effect. We also observe that the function *z*(*v*) is remarkably similar in the two models (independently of the dispersal kernel employed), as demonstrated in Fig. 2b where 1/*z* is shown as a function of 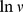. In particular, both models are fairly well described by the fitting formula [27].

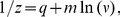
(2)where the constants *q* and *m* are model-dependent.

For the MVM (with NN-dispersal), some mathematical results are available for 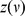. Durrett and Levin [23] (see also [25]) provided the asymptotic estimate 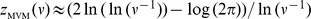, which is consistent with the fitting formula (2), but for a very slow variation of the slope *m* due to the 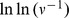 term. The specific values of *m* and *q* obtained by the numerical simulations are slightly different from those implied by the asymptotic estimate (see Ref. citePigolotti2009 for a detailed discussion). For the SSM, as described in Appendix S3, we derived the approximate asymptotic formula

**Figure 4:**
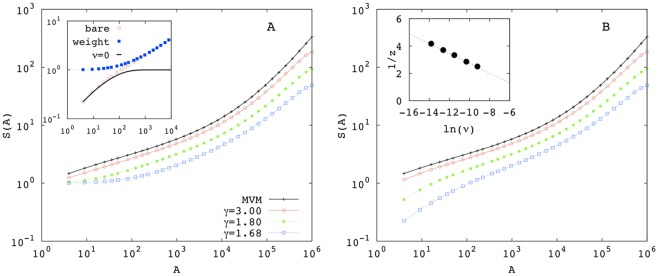
SARs for the MCP for various 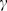 as labeled: (a) with weighted averages Eq. (5) and (b) with bare averages Eq. (4). Inset of (a): for 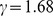, comparison among bare and weighted averages for 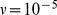 with the bare average for *v* = 0. Notice the flattening of the weighted curve at short scales and the equivalence of both averages at larger scales. Inset of (b): 1/*z* vs 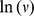, the straight line shows formula Eq. (2) with fitted values 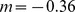 and 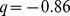. The exponents *z* were estimated as the minimum of the local slopes of the species-area curves.



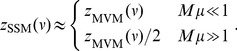
(3)Consistently with our numerical results, the above estimate predicts that in the limit of large local population sizes (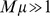) the species-area curves of the SSM are shallower than those of the voter model, which are recovered in the limit of small local population sizes (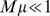). We also mention that the fitting formula Eq. (2) is also compatible with the result of an exactly solvable variant of the neutral model [41].

The following two sections focus on the SSM and the MCP, to further elucidate the importance of local community size and habitat saturation on the variability of SAR curves.

### Multispecies Stepping Stone Model

Sensitive variations of *z* are indeed observed by changing *M* and 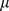, for fixed speciation to migration ratio 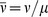. In particular, the exponent *z* decreases with 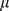 and *M* and seems to be mainly determined by their product 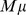, as shown in Fig. 3 for two different values of 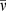.

For 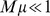 the exponent *z* approaches the corresponding value in the MVM, while at large 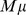 the exponent decreases in a sigmoidal fashion and displays a tendency towards a different asymptotic value. These two limits correspond to very different regimes. When 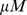 is very small, sites have a small local population and species (individuals) exchanges among sites are rare: most sites are not able to sustain diversity and contain only one species (i.e. in this regime local fixation dominates). In this limit, the SSM reproduces MVM behavior with the on-site mono-dominant community in the former playing the role of a single individual in the latter. Conversely, when 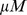 is very large and *v* is very small, the large local community size (buffering local extinctions and fixations) and the frequent exchanges among sites allow each site to host a large number of species on average. A further consequence is that each species will be statistically represented in a similar way at each site of a large region. Also, distant sites can now host many common species. This leads to shallower species-area curves, and thus to the smaller *z* values shown in Fig. 3. It is worth remarking that shallower SARs do not necessarily mean lower diversity as, for 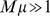, the prefactor in front of the power-law (1) can be very large (not shown here).

Remarkably, the above qualitative argument can be supported by analytical estimates, see Eq. (3). By generalizing the calculation of Durrett and Levin [23] (see also [25]), we have been able to estimate that, for 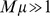, one should observe 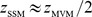 (see Appendix S3 for details). The numerical results of Fig. 3 display the correct tendency: for the largest values of 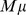 we could explore, we observe that *z* is reduced by a factor 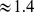 with respect to 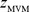. This behavior is also confirmed for varying values of 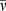 (not shown). It would be very interesting to explore the (numerically costly) larger values of 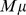 to test the theoretical prediction.

We close this section observing that in the right panel of Fig. 3 we also show the value of the exponent *z* obtained by using the square kernel for both the MVM and the SSM (we only show the value for 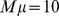 here as it is already close to the saturation regime). As already mentioned while the MVM with the square kernel is not far from the values of the exponent obtained with the SSM with NN-dispersal, still the exponent for the SSM with square kernel is sensitively smaller, confirming the robustness of the effect of increasing the local population size.

### Multispecies Contact Process

At fixed speciation rate and varying the birth-to-death ratio 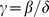 of the MCP, we can inspect how the level of habitat saturation affects SAR-curves. For 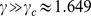, the habitat is close to saturation, as the density of occupied sites approaches 1, and the MCP is equivalent to MVM. Indeed, as shown in Fig. 4, the curves generated by the two models are essentially coincident already for 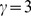.

For highly non-saturated habitats, i.e. for smaller values of 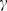, larger and larger areas with very few (or zero) individuals become more and more probable. In this regime, SAR curves display a strong dependence on the choice of the sampling procedure, as we illustrate here with two examples.

The first procedure, that we dub “bare average”, consists in ignoring the non-saturation of the habitat and thus averaging over many samples of fixed area *A*, regardless of the number of individuals they host:
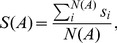
(4)where *s_i_* is the number of distinct species in sample *i* and *N*(*A*) is the total number of samples of area *A*. With this procedure, *S*(*A*) will be inevitably affected by the spatial variations of the density of individuals. For instance, for *A* = 1 (i.e. on a single site), *s_i_* = 1 or *s_i_* = 0 so that *S*(1), as given by Eq. (4), reduces to the average density.

A second and more appropriate choice is to put less weight on areas with a smaller number of individuals, where the number of observed species is statistically biased to be small. In particular, by denoting with *n_i_* the number individuals present in the area *a_i_*, we define the “weighted average” (which was used in Fig. 1) as
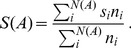
(5)


SAR curves for different values of 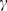 are shown in Fig. 4a and 4b for weighted and unweighted averages, respectively. For large 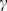, the dependence on the averaging protocol (if any) is mild, while it is strong for small values of 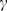. The strongest effects are observed at small areas, where bare averages Eq. (4) are influenced the most by local densities. This effect is demonstrated in the inset of Fig 4a, where the black line is simply the fraction *P*(*A*) of regions of area *A* occupied by at least one individual (of any species). For small areas, the weighted average becomes very shallow without any signature of power-law behavior (see inset of Fig. 1). Conversely, the bare average almost coincides with *P*(*A*), demonstrating its lack of sensitivity to the presence of more than one species at small scales. At larger scales, where 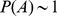, the two averages coincide.

In contrast with weighted averages and similarly to the other models in Fig. 1, the local slopes of bare averages display a parabolic intermediate range with a well defined minimum, from which we can extract an estimate of exponent *z*. Fig 4b shows 1/*z* as a function of 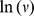 for 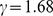; formula (2) fits well these data, yielding values of *z* being larger that those for the MVM and the SSM. However, as detailed in Appendix S5, in this case the interpretations of these exponents is problematic as the power-law can be induced by the spatial fluctuations of the density of individuals rather than by species distribution.

## Discussion

In this paper, we have studied the effect of changing the level of habitat saturation and the local population size on spatial neutral models. We have shown that species-area laws quantitatively depend on these ecological features, which go beyond the previously explored variations due to long-ranged dispersal kernels [28]. Spatially explicit neutral models thus seem to be much richer in structure than spatially implicit ones, where the species-abundance distribution seems to be insensitive to implementation details. Moreover, the observed variations of SARs suggest that spatial neutral theories can explain part of the variability of the exponent *z* observed in nature.

In spatially explicit neutral models, SAR curves typically display a range of scales where they are well approximated by the power law (1), in particular at small scales for NN-kernels and at intermediate scales for finite range kernels. We have shown that, generally, the inverse exponent 1/*z* is very well described as a linear function of 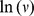, the logarithm of the rate of the introduction of new species *v*. The same kind of behavior was analytically confirmed in an exactly solvable neutral model [41]. However, the coefficients of this linear relation and thus the actual value of *z* are sensitive to the ecological factors implemented in the model. The logarithmic behavior is a general and robust feature related to two common features of all neutral models discussed here: species originate with one individual (point speciation mode) and then diffuse in space. Altering the speciation mechanisms in spatially implicit models affects some aspects of SADs [43]–[45]. It would be interesting, in the future, to study how different speciation modes reflect into the spatial variation of biodiversity, a program which just started in the context of spatial models (see [38] and references therein). In the context of the models considered in this paper, assuming that variations in *z* are caused by *v* variability among different taxa, amounts to say that the diversification rate per capita per generation increases at increasing body mass [27]. While this possibility cannot be completely ruled out (owing mostly to the difficulty of estimating such rates), organisms such as bacteria are characterized by high mutation rates and genetic plasticity, rather suggesting a higher rate of differentiation even when considered at the individual level.

Relaxing the hypothesis of *habitat saturation* –as occurs in the multispecies contact process– does not greatly modify the behavior of species-areas curves with respect to the saturated case –i.e. the multispecies voter model–, unless the habitat becomes too fragmented. In the latter case, species-area curves strongly depend on the sampling procedure. In particular, using “weighted averages” (which weight to the sampled area proportionally to the population it hosts) the contact process generates SARs convex in log-log scale, with no clearly detectable power-law regime.

Conversely, allowing for variations in the *local population size* –as occurs in the stepping stone model– leads to a monotonic decrease of the exponent *z* as the number of individuals per site *M* is increased. For large values of *M* we numerically found a reduction in the exponent *z* up to a factor 1.4 with respect to the *M* = 1, voter model, value. Our analytical estimate (3) suggests that this factor can be actually larger (up to a factor 2) when the community size *M* becomes very large. This is a quite remarkable result in view of the fact that microorganisms, for which a description in terms of very large local communities is appropriate [33], do actually spatially structure themselves with shallow taxa-area laws [11], [12] characterized by smaller values of *z*. For instance, a recent review [13] reports results for salt-marsh bacteria, marine diatoms, arid soil fungi, and marine ciliates and shows that, in contiguous habitats, *z*-values for all these categories are roughly the half as those for larger animals and plants, in surprisingly good agreement with our results. It is worthwhile to remark that the reduced diversification of microorganism has been sometimes ascribed to the possibility of long distance dispersal [33]. However, numerical results of Ref. [28] show that distant dispersal events increase rather than decrease the local slope in the intermediate regime and thus the value of the exponent *z* (while the local slope at larger scales decreases). Therefore, it is unlikely that large distance dispersal events can –in the absence of additional mechanisms– account for the observed small value of the exponents in microbial communities.

More generally, the spatial variation of biodiversity observed in the stepping stone model suggests an explanation for the observed “cosmopolitan” behavior of microorganisms [33], [46], [47], where relatively small areas are found to contain significant fractions of the species known in the entire globe. This phenomenon is remarkably well captured by the SSM where, upon increasing the local population size, each site tends to contain a considerable fraction of the entire biodiversity found in a large area. In conclusion, the results obtained with the stepping stone model add mathematical support, within the neutral theory framework, to large population sizes being one of the mechanisms for the shallower SAR curves observed in microorganisms (as put forward by Fenchel and Finally [33], [46], [47]). Specifically, the SSM shows that having a large population size, within a well mixed patch, provides a buffer to local extinctions and enhances the local fixations time, making inter-patch migration more effective and the whole ecosystem closer to a panmictic population with, consequently, a lowered spatial diversification.

## Supporting Information

Appendix S1 **Here we briefly summarize the dual representation of the multitype voter model.**
(PDF)

Appendix S2 **This Appendix extends the dual representation to the Stepping Stone model and briefly sketches its numerical implementation.**
(PDF)

Appendix S3 **This Appendix presents the derivation of an asymptotic formula for the exponent** ***z*** **for the Stepping Stone model, after recalling the main idea developed for the voter model.**
(PDF)

Appendix S4 **Herein the numerical scheme used to simulate the multitype contact process is briefly explained.**
(PDF)

Appendix S5 **This Appendix shows how spurious results can come out for the species-area relationship in the case of the contact process, when an improper average is used.**
(PDF)
