# Supplementary material for: What Ecological Factors Shape Species-Area Curves in Neutral Models?
Source: PLoS One. 2012 Jun 4;7(6):e38232. doi: 10.1371/journal.pone.0038232 (PMC3366919; doi:10.1371/journal.pone.0038232)
Supplement: Appendix S1 — Here we briefly summarize the dual representation of the multitype voter model. (PDF) [file pone.0038232.s001.pdf]

Appendix S1

Dual representation of voter and stepping stone models

To compute SAR-curves, one needs to generate several spatial configurations of the different models and average over them. Generating such configurations amounts to identify, at a certain (long) time t, to which species the individuals residing at each lattice site belong to. A straightforward algorithm is to evolve the system from time 0 to time t according to the dynamical rules described in the main text. For the multispecies voter model (MVM) and stepping stone model (SSM) an alternative strategy exists, that is to employ the so-called dual dynamics. This idea stems from the work of Liggett [1], who recognized the relation between the voter model without speciation ($\nu = 0$) and a system of coalescing random walkers moving backward in time, and from the coalescent theory introduced by Kingman [2, 3] in the context of population genetics.

Let us start by briefly recalling the dual representation for the voter model. In the voter model an individual creates a replica of itself at a randomly chosen nearest neighbor site. In the backward picture, the sequence of *ancestors* of any given individual, existing at time t, is seen as a random walk moving backward in time on the lattice. If, at a certain time $s < t$, two random walkers meet at a site, the corresponding two individuals have a common ancestor and thus belong to the same species. Hence, for times, $t' < s$ the two walkers *coalesce* into one. As a consequence, as time evolves backward, the number of walkers is progressively reduced. Introducing a non-vanishing speciation rate, ν, corresponds to annihilating random walkers at the same rate, i.e. to terminating backward paths. In this way, the voter model with speciation or multispecies voter model (MVM) turns out to be *dual* to a system of diffusing and annihilating random walkers moving backward in time. The dual representation allows for understanding some properties of the MVM and SSM in terms of diffusive processes [4], and for deriving analytical predictions (see Appendix S3). Moreover, it suggests efficient algorithms for numerical simulations. For the MVM, details of such algorithms can be found in [5–8], while details of our own results for the dual theory of the SSM, together with a description of an efficient algorithm for computer simulations, are described in the next subsection.

References

1. Holley R, Liggett T (1975) Ergodic theorems for weakly interacting infinite systems and the voter model. Annals of Probability 3: 643–663.

2. Kingman JFC (1982) The coalescent. Stochastic processes and their applications 13: 235–248.

3. Kingman JFC (1982) On the genealogy of large populations. Journal of Applied Probabability 19: 27–43.

4. Durrett R, Levin S (1996) Spatial models for species-area curves. Journal of Theoretical Biology 179: 119–127.

5. Chave J, Muller-Landau H, Levin S (2002) Comparing classical community models: theoretical consequences for patterns of diversity. American Naturalist 159: 1–23.

6. Rosindell J, Cornell S (2007) Species–area relationships from a spatially explicit neutral model in an infinite landscape. Ecology Letters 10: 586–595.

7. Rosindell J, Wong Y, Etienne R (2008) A coalescence approach to spatial neutral ecology. Ecological Informatics 3: 259–271.

8. Pigolotti S, Cencini M (2009) Speciation-rate dependence in species-area relationships. Journal of Theoretical Biology 260: 83–89.
