# Supplementary material for: What Ecological Factors Shape Species-Area Curves in Neutral Models?
Source: PLoS One. 2012 Jun 4;7(6):e38232. doi: 10.1371/journal.pone.0038232 (PMC3366919; doi:10.1371/journal.pone.0038232)
Supplement: Appendix S2 — This Appendix extends the dual representation to the Stepping Stone model and briefly sketches its numerical implementation. (PDF) [file pone.0038232.s002.pdf]

# Appendix S2

## Dual algorithm for the Stepping Stone Model

The dual process of the SSM is constructed by following the same idea as that for the MVM (Appendix S1). The main difference is that in the SSM each site can host $M$ individuals, and reproduction events can happen either at the same site with probability $1-\mu$ or at a neighbor site with probability $\mu$. In the backward picture, each site contains $M$ compartments, hosting –at maximum– one walker each. The dynamics starts by placing a walker at each compartment of a $L \times L$ square-lattice, resulting in $N_w = M \times L^2$ walkers. As for the MVM, the walkers move backward in time coalescing and annihilating. The only difference here is that coalescence occur only if the walkers end up in the same compartment within the same site.

More specifically, the dual algorithm is implemented as follows. At each time step, a walker is randomly picked, and:
**(i)** killed with probability $\nu$. Then, the number of species $S$ (set to zero at the beginning) is incremented by one unit and assigned to the dead walker, which is removed from the pool of alive walkers ($N_w$ decreased by one).
**(ii)** with probability $1-\nu$, the walker moves:

**(iia)** with probability $1-\mu$, it moves to a randomly chosen compartment among the other $M-1$ belonging to the same site. If it was occupied, coalescence takes place, and one of the two coalesced walkers is removed from the list of alive walkers ($N_w$ decreased by one).

**(iib)** with probability $\mu$, it moves to a randomly chosen compartment of any of the neighbor sites. If such compartment was occupied, coalescence occurs as in **(iia)**.

Each simulation ends when a single walker remains alive ($N_w = 1$); this is then killed as in **(i)**. Then, the stored information about the coalescing and annihilating events for each walker allows the genealogy of each individual to be reconstructed and thus we can assign a species to each individual, compartments by compartments and site by site.

The MVM corresponds to $M = \mu = 1$. With $M = 1$ and $\mu < 1$, events **(iia)** correspond to time steps in which walkers do not move, leading to a time rescaling with respect to the $\mu = 1$ case. This clarifies why the effective speciation-to-diffusion ratio, $\nu/\mu$, is the appropriate parameter to compare the SSM with the MVM.

Simulations based on the backward dynamics present a number of advantages. As both annihilation and coalescence events decrease the number of walkers, the computation speeds up also when $\nu$ is very small (where the bottleneck becomes the speed of the random number generator). When there are many alive walkers (i.e. at the beginning), the slowest operation is to search for the collision/coalescence partner **(ii)**; this search is made efficient by means of a look-up table. Moreover, simulations are free of finite-size boundary effects. As walkers can move in the whole plane, they effectively sample a portion –of size $L \times L$– of an infinite system. This means that, to explore the power-law intermediate regime of SAR-curves, there is no need to consider huge systems to avoid finite-size effects, at variance with the forward dynamics (as discussed in Appendix S4 for the MCP). Finally, there is no need to wait for a statistically steady state to establish, as is the case for the forward dynamics. In the dual representation, each simulation generates, by construction, a statistically stationary configuration.

As annihilating and coalescing events are independent, one could in principle perform simulations at $\nu = 0$, and perform killing (speciation) a posteriori by pruning the list of coalescences. In this way one could use the same realization of the coalescing random walks to increase the statistics by averaging over different speciation histories. This is a standard procedure in population genetics [1], and can also be implemented in this context [2]. Here we did not implemented such a procedure owing to its memory requirements for large $M$ values and large lattice sizes.

# References

1. Gillespie J (2004) Population genetics: a concise guide. Baltimore, Maryland: The Johns Hopkins University Press.

2. Rosindell J, Wong Y, Etienne R (2008) A coalescence approach to spatial neutral ecology. Ecological Informatics 3: 259–271.
