# Supplementary material for: What Ecological Factors Shape Species-Area Curves in Neutral Models?
Source: PLoS One. 2012 Jun 4;7(6):e38232. doi: 10.1371/journal.pone.0038232 (PMC3366919; doi:10.1371/journal.pone.0038232)
Supplement: Appendix S3 — This Appendix presents the derivation of an asymptotic formula for the exponent z for the Stepping Stone model, after recalling the main idea developed for the voter model. (PDF) [file pone.0038232.s003.pdf]

Appendix S3

Estimates for z in the MVM and the SSM

The backward dynamics can also be used to obtain analytical estimates for z. Before deriving the new results for the SSM, we sketch the original idea developed for the MVM in Refs. [1,2].

The timescale for a new species to appear, i.e. the typical time for walkers to annihilate in the dual representation, is $\tau = 1/\nu$. Walkers diffuse in space and thus after a time t, on average, they move a distance $\propto t^{1/2}$ from their origin. This means that, associated with τ, there is a characteristic spatial scale $\xi = \sqrt{\tau} = \nu^{-1/2}$. Given a sample of area $\xi^2 = \nu^{-1}$, the number of species present in the sample $S(\xi^2)$ is given by the total number of annihilated walkers, which can be estimated as follows. In a two-dimensional system of coalescing walkers, with short range dispersal and without annihilation, the density of walkers decreases asymptotically as [3]

$$\rho(t) \approx \frac{\ln t}{\pi t} \,. \tag{S3-1}$$

The annihilation rate at time t can be estimated as the annihilation rate per walker, ν, times the average number of walkers at time t, i.e. $\xi^2\rho(t)$. Its time integral gives the total number of annihilations (the distribution of annihilations is Poissonian), so that

$$S(\xi^2) \sim \nu\xi^2 \int_{t_0}^{\tau=\xi^2} dt\, \rho(t) \sim \frac{\ln^2(\xi^2) - \ln^2(t_0)}{2\pi} \tag{S3-2}$$

where t_0 is the time at which the asymptotic scaling relation sets in. We assume $\tau = \xi^2 \gg t_0$ and drop the dependence on t_0 in the above expression. Moreover, the number of annihilations happening for times larger than τ is bounded and can be neglected [2]. Finally, the estimate of Eq. (S3-2) assumes the number of annihilations being negligible compared to the number of coalescences, i.e. ν is very small.

Postulating the scaling form $S(A) \approx N(A) \sim A^z$ for $A \in [1 : \xi^2]$ and using the fact that in an area $A = 1$ there is only one species, one obtains [1]

$$z = \frac{\ln S(\xi^2) - \ln S(1)}{\ln \xi^2} \sim \frac{2\ln(\ln(\nu^{-1})) - \ln 2\pi}{\ln(\nu^{-1})} \approx \frac{2\ln\ln(\nu^{-1}))}{\ln(\nu^{-1})} \,. \tag{S3-3}$$

We recall that this estimate captures the observed logarithmic dependence of z on ν, but it is unable to match the proportionality constant computed in numerical simulations [4].

Let us now discuss the SSM. In this case, two different regimes $M\mu \leq 1$ and $M\mu >> 1$ should be distinguished. In the former, all walkers at any given site typically coalesce intra-site before having the time to jump to neighboring sites, so that essentially no inter-site coalescences occur before all walkers at any site coalesce into just one. Once this has happened, the system becomes voter-like, and one can repeat the calculation above, but with the diffusion time replaced by an effective one being $t\mu$. Consequently, one retrieves the result (S3-3) with ν replaced by the speciation to migration ratio: $\bar{\nu} = \nu/\mu$.

In the opposite limit $M\mu >> 1$, intra-site coalescence is limited by diffusion and by the size of the local population M (after the initial stage, when the density of walkers has decreased, many compartments at the same site will be empty so that when two walkers land on the same site, the probability for them to coalesce is very small). Since walkers wander for long times before coalescing, we make the simplifying assumption that all couples of walkers within an area $\xi^2 \approx \bar{\nu}^{-1}$ have the same chance to coalesce, regardless of their initial separation. In other terms, we assume that the population living patches of size smaller than ξ^2 is well mixed. Consequently, we use the mean field formula [5] according to which, in the absence of annihilation, the density of walkers decay as

$$\rho(t) \sim t^{-1} \tag{S3-4}$$

as opposite to Eq. (S3-1). In this case, the equivalent of Eq. (S3-2) becomes

$$S(\xi^2) \sim \nu M \xi^2 \int_{t_0}^{\tau=\xi^2} dt\, t^{-1} \sim M\mu[\ln(\xi^2) - \ln(t_0)]\,. \tag{S3-5}$$

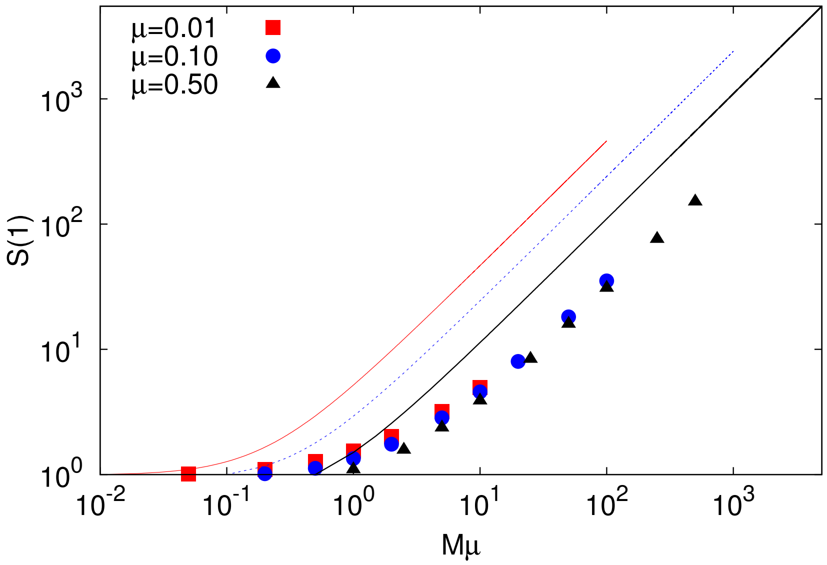

Figure S3-1. Average number of species in a single site of the SSM with $\bar{\nu} = \nu/\mu = 10^{-8}$ (parameters as in Fig. 4 of the main text) as a function of $M\mu$, for $\mu = 0.01, 0.1$ and 0.5 (as labeled). The solid lines are obtained from Eq. (S3-6), where the colors correspond to the values of μ chosen in the simulations. Numerical simulation and theory display a linear behavior for large $M\mu$. The quantitative agreement between the prefactors decreases as μ is decreased as low values of μ generate correlations in the sample not captured by formula (S3-6). Finally, notice that simulations suggest $S(1)$ being a function of $M\mu$ only while in the sampling formula $S(1)$ at fixed $M\mu$ still shows a dependence on μ, see text for a discussion.

In order to compute z, we also need an estimate for $S(1)$, which in this case is not fixed to be 1 like in the voter model. As we are assuming the population in an area ξ^2 to be well mixed, we can think of a single site as a sample of M individuals from this population and make use of Ewens' sampling formula [6–8]. As derived, e.g., in [7] we have that

$$S(1) = \sum_{j=0}^{M-1} \frac{\theta}{\theta + j} \approx M\mu \log(1 + \mu^{-1}) \tag{S3-6}$$

where θ is the product of the panmictic population size ($M\xi^2$) times the speciation rate ν, i.e. $\theta = M\xi^2\nu = M\mu$, and the last expression has been derived by approximating the sum by an integral. Proceeding as in Eq. (S3-3), using Eq. (S3-5) and Eq. (S3-6), we find

$$z \sim \frac{\ln(\ln(\bar{\nu}^{-1})) - \ln C}{\ln(\bar{\nu}^{-1})} \approx \frac{\ln(\ln(\bar{\nu}^{-1}))}{\ln(\bar{\nu}^{-1})}, \tag{S3-7}$$

where $C \approx \log(1+\mu^{-1})$. Notice how, in the limit of small $\bar{\nu}$, z is a factor 2 smaller than the prediction of Eq. (S3-3) for the voter model.

We conclude by remarking that the panmictic behavior of the SSM is a rigorous result when periodic boundary conditions are implemented on a square of side L and $M\mu \gg \ln(L)$, as shown in [9]. Conversely, in the open boundary condition case considered here it must be taken as an approximation, whose accuracy may depend on the spatial scale. We tested this approximation by comparing the estimate of the average number of species in one site from numerical simulation with the prediction based on the Ewens' sampling formula. Results are presented in Fig. S3-1, showing that the linear behavior in $M\alpha$ is well predicted by formula (S3-6), apart from a difference in the prefactor. In particular, simulations suggest a prefactor $C \approx 0.3$, which seems to be independent of μ (so that $S(1)$ becomes a function of $M\mu$ only), while the estimate of Eq. (S3-6) predicts a constant dependent on μ which deviates more from the numerical results as μ is decreased. The reason of this deviation can be ascribed to the effect of inter-site coalescence at smaller values of μ, reducing the number of species compared to the theory, i.e. compensating the increase of M (at $M\mu$ fixed). Another way of seeing it is that individuals belonging to the same site at low μ constitute a correlated sample of the population. Conversely, in Ewens prediction (S3-6) when M increases (at fixed $M\mu = \theta$) it means that the sample size increases leading, obviously, to a larger number of species.

The issue above demonstrates a problem common also to the estimates for the MVM: quantitative agreement between theory and simulations can be compromised by hard-to-estimate constants, whose contribution becomes irrelevant only for inaccessibly small values of ν (where the only relevant contribution is the $\ln\ln\nu$ term).

References

1. Durrett R, Levin S (1996) Spatial models for species-area curves. Journal of Theoretical Biology 179: 119–127.

2. Bramson M, Cox JT, Durrett R (1996) A spatial model for species area curves. Annals of Probability 24: 1727–1751.

3. Bramson M, Griffeath D (1980) Asymptotics for interacting particle systems on Z^d. Probability Theory and Related Fields 53: 183–196.

4. Pigolotti S, Cencini M (2009) Speciation-rate dependence in species-area relationships. Journal of Theoretical Biology 260: 83–89.

5. Peliti L (1986) Renormalisation of fluctuation effects in the A+ A to A reaction. Journal of Physics A: Mathematical and Geneneral 19: L365-L367.

6. Ewens W (1972) The sampling theory of selectively neutral alleles. Theoretical Population Biology 3: 87–112.

7. Tavaré S (1997) Multivariate Ewens distribution. In: Discrete Multivariate Distributions. Wiley, New York.

8. Durrett R (2008) Probability models for DNA sequence evolution. New York: Springer Verlag.

9. Cox J, Durrett R (2002) The stepping stone model: New formulas expose old myths. Annals of Applied Probabability 12: 1348–1377.
