# Supplementary material for: What Ecological Factors Shape Species-Area Curves in Neutral Models?
Source: PLoS One. 2012 Jun 4;7(6):e38232. doi: 10.1371/journal.pone.0038232 (PMC3366919; doi:10.1371/journal.pone.0038232)
Supplement: Appendix S4 — Herein the numerical scheme used to simulate the multitype contact process is briefly explained. (PDF) [file pone.0038232.s004.pdf]

Appendix S4

Numerical implementation of the MCP

The contact process is a self-dual model [1], meaning that its dual representation is the contact process itself. Therefore, duality does not provide any useful help in this case, and one needs to resort to standard forward-time simulations. Here below we briefly sketch the algorithm we used and discuss some related issues.

We implemented a multispecies generalization of standard algorithms for the contact process [2]. A $L \times L$ two-dimensional lattice with periodic boundary conditions is initialized by placing and individual at each lattice site; its species is labeled by a positive integer s ($s = 0$ means that the site is empty). Initially, a single species occupies the whole system. We keep track of occupied sites in a list, containing $N_{occ} = L^2$ entries at time $t = 0$. At each step, time is incremented by L^2/N_{occ} and a random individual (in the list of non-empty sites) is chosen:
(i) with probability $\delta/(\beta+\delta)$ it is killed and removed from the list (N_{occ} decreases in one unit)
(ii) with probability $\beta/(\beta + \delta)$, reproduction at a randomly chosen neighbor site is attempted:

(iia) if the chosen neighbor site was non-empty, reproduction is unsuccessful, and the state of the system does not change

(iib) if it was empty, reproduction is successful (N_{occ} increases in one unit)

(iib.1) with probability $1 - \nu$ the site becomes occupied by an individual from the parent species and is added to the list of occupied sites;

(iib.2) with probability ν the newborn mutates from the parent, giving birth to a new species. A new species-label is created by increasing in unity the largest existing one, and it is assigned to this site (which, on its turn, is added to the list of non-empty sites).

When $\gamma = \beta/\delta > \gamma_c$, the system evolves to a dynamical equilibrium, with new species appearing and older ones becoming eventually extinct. The number of extant species is monitored. Once equilibration of the density of non-empty sites is reached, on longer time scales (on the order of ν^{-1}) also the number of extant species equilibrate fluctuating around a mean value. After equilibration, configurations of the system are periodically sampled, and used to compute the SAR. Notice that, in order to have statistically independent measurements, the sampling interval should be also on the order of ν^{-1}. Simulations are terminated when enough statistics have been collected.

At variance with the backward algorithm for the SSM and MVM, here boundaries play and important role, requiring rather large lattices to avoid finite size effects. In particular, L must be larger than $1/\sqrt{\nu}$, which sets (similarly to the SSM and MVM) an approximate scale on which individuals are expected to diffuse before speciation. If L is taken too small, the measured effective value z underestimates the true one. By comparing SARs obtained at equal parameters and different system sizes, we determined that a safe choice to neglect finite size effect is $L^2 = 10/\nu$. We also checked that for this system size and large values of γ the results for the MVM are recovered.

Consequently, at decreasing ν simulations become more and more demanding both because a larger system size is required and because relevant time scales become slower and slower. Owing to these limitations, we could not simulate systems with ν smaller than 10^{-6} with enough statistics.

References

1. Liggett T (1985) Interacting particle systems. Berlin: Springer Verlag.

2. Marro J, Dickman R (1999) Nonequilibrium Phase Transitions in Lattice Models. Cambridge: Cambridge University Press.
