# Supplementary material for: What Ecological Factors Shape Species-Area Curves in Neutral Models?
Source: PLoS One. 2012 Jun 4;7(6):e38232. doi: 10.1371/journal.pone.0038232 (PMC3366919; doi:10.1371/journal.pone.0038232)
Supplement: Appendix S5 — This Appendix shows how spurious results can come out for the species-area relationship in the case of the contact process, when an improper average is used. (PDF) [file pone.0038232.s005.pdf]

Appendix S5

Behavior of bare average for the MCP at low densities and low speciation rates

We discuss in detail how approaching the critical birth-to-death rate ratio, $\gamma \to \gamma_c$, SARs measured using the bare average (Eq. (4) of the main text) are mostly determined by spatial fluctuations of the density of individuals rather than by species diversity.

For $\gamma = \gamma_c$, the set of occupied sites constitutes a fractal set — with fractal-dimension $d_F < d$ — embedded in the d-dimensional space (see, e.g. [1]). In this limit, the coarse-grained density $\rho(A)$ of regions of area A occupied by at least one individual grows as $\rho(A) \sim A^{d-d_F}$. As a consequence, when ν is very small and bare averages are chosen, one has $S(A) \approx \rho(A) \sim A^{d-d_F}$ for small areas, leading to an estimate $z = d - d_F$.

Unfortunately, in two dimensions $d = 2$, it is very hard to verify this prediction, due to the difficulties in simulating MCP close to γ_c and for small values of ν. Here, we demonstrate this effect in the numerically simple one-dimensional case. In $d = 1$, one has $d_F \approx 0.75$ (see, e.g. [1]), and the previous argument predicts $z = d - d_F \approx 0.25$. This is confirmed in Fig. S3-1 where we show $S(A)$ for $\nu = 0, 10^4$ and 10^{-5} for $\gamma_c - \gamma = 10^{-4}$.

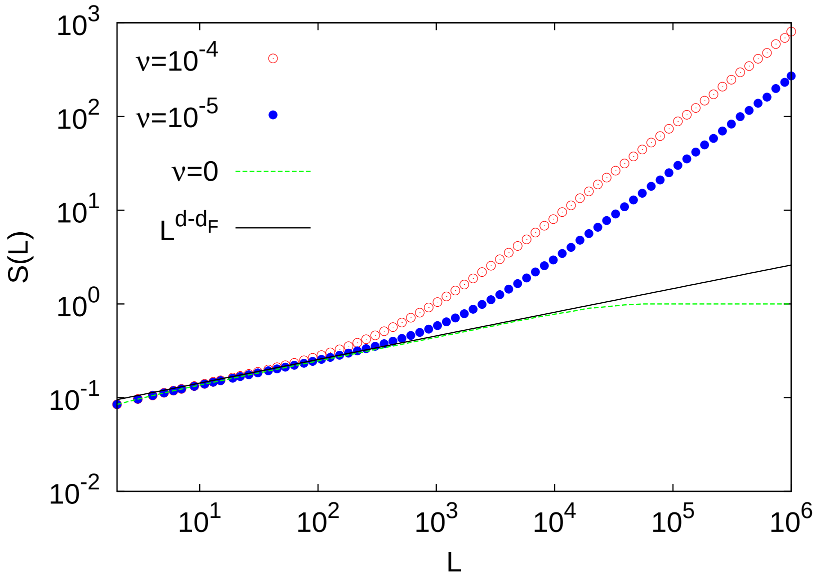

Figure S3-1. Species-length relationship for the 1D contact process close to the critical birth-to-death rate ratio, $\gamma_c - \gamma = 10^{-4}$ (in $d = 1$ $\gamma_c = 3.29785(2)$) for $\nu = 10^{-4}$ (red symbols) and 10^{-5} (blue symbols), in a lattice of size 10^6. Bare averages (Eq. (4) of the main text) are employed to compute the number of species $S(L)$ over segments of length L. At $\gamma_c - \gamma = 10^{-4}$ criticality becomes evident: occupied sites live on a fractal set of dimension $d_F \approx 0.75$, which implies a spurious power-law behavior $S(L) \sim L^z$ with $z = d - d_F \approx 0.25$, as shown by the black straight line. Also shown for comparison is the bare average with $\nu = 0$ (only one species). Notice how this coincides with the $\nu \neq 0$ SLR for L short enough, confirming that the power-law has a pure geometrical origin.

Finally, we remark once more that in the framework of SARs this regime must be considered as an artifact induced by bare averages, in the sense that the resulting power law does not contain information

about species diversity. This is made more clear in the figure, where the same power law is observed for $\nu = 0$ where only one species is present.

References

1. Marro J, Dickman R (1999) Nonequilibrium Phase Transitions in Lattice Models. Cambridge: Cambridge University Press.
